# Special Issue: Vaccines against Antibiotic-Resistant Bacteria: From Bench to Bedside

**DOI:** 10.3390/vaccines12010068

**Published:** 2024-01-09

**Authors:** Mireia López-Siles, Andrés Corral-Lugo, Michael J. McConnell

**Affiliations:** 1 Microbiology of Intestinal Diseases, Biology Department, Universitat de Girona, 17003 Girona, Spain; 2 Intrahospital Infections Unit, Reference and Research Laboratory in Resistance to Antibiotics and Infections Related to Healthcare, National Centre for Microbiology, Instituto de Salud Carlos III (ISCIII), 28029 Madrid, Spain; 3 Department of Biological Sciences, University of Notre Dame, Notre Dame, IN 46556, USA

The emergence and global dissemination of bacterial strains from numerous species with resistance to multiple antibiotic classes has increased in recent years, both in the healthcare and the community setting. Currently, antimicrobial resistance is a major threat to global public health, which is projected to worsen in the years to come.

To overcome this situation, in addition to existing approaches, novel strategies to fight against the most concerning species, including both Gram-positive and Gram-negative bacteria, are required. In this context, vaccination has been proven as an effective approach for preventing and reducing bacterial infections that cause diphtheria, tetanus, meningitis, bubonic plague, and anthrax, among others. Therefore, according to the World Health Organization’s recommendations, the research, development, and implementation of vaccines are particularly necessary, since vaccination can help to limit the spread of antibiotic resistance. In this context, this Special Issue comprises four original research studies and a review, elucidating the potential for vaccines to contribute to reducing the burden of disease of infections caused by antibiotic-resistant bacteria.

The article by MatRahim et al. (contribution 1) reports the development of a potential oral vaccine against *Acinetobacter baumannii.* As *Bacillus subtilis* is considered a “Generally Recognized As Safe” organism approved by the US FDA for human consumption, the authors developed recombinant spores that display TonB-dependent receptor proteins from *A. baumannii*. In this preclinical study, the administration of three doses of the recombinant spores is proven to elicit a protective immune response in mice. Specifically, IgG and IgA levels in blood and intestinal secretion, complement-mediated bacterial killing, and opsonophagocytic assays were assessed. Although the extent of the resulting immune response varies according to the TonB-dependent receptor protein expressed on the spores, this study provides *in vitro* evidence that the vaccine-induced immune response may be able to protect against multidrug-resistant *A. baumannii* clinical isolates recovered from different types of infections.

Two articles in this collection focus on another pathogen of critical concern, namely *Pseudomonas aeruginosa.* In the first contribution, Sheweita et al. (contribution 2) report for the first time the use of bacterial ghosts from this species as a vaccine. Chemically induced bacterial ghosts were orally administered, and the efficacy of protection against ulcer infection was assessed in diabetic rats. The authors demonstrate that this approach generates both humoral and cell-mediated immune responses and may be a strategy for the prevention of skin wound infection. In the other contribution, Hamad et al. (contribution 3) combined both *in silico* and *in vivo* tests to design a recombinant protein vaccine targeting the iron acquisition proteins of the same species. Since iron is essential for bacteria to establish infection, using reverse vaccinology, the authors performed an exhaustive screening of previously published expression data and identified the hemphore HasAp of *P. aeruginosa* as the most suitable candidate. Subsequent immunization experiments with recombinant HasAp alone or with naloxone adjuvant did not provide sufficient protection against infection. Future studies may include other immunization regimens, models of infection, immunoadjuvants, and/or combinations with other iron acquisition proteins to improve protection efficacy.

Malik et al. (contribution 4) performed an *in silico* design of a multi-epitope vaccine for *Brucella melitensis*, the agent responsible for brucellosis. Several computational methods were used, including pathogen core proteome screening for the identification of suitable vaccine candidates and subsequent epitope screening for antigenicity, allergenicity, and water solubility. The vaccine construct was further assessed for binding efficacy with immune cell receptors, and it proved effective in inducing immune responses. Therefore, this approach could be helpful for experimental researchers to improve the success rates of trials for vaccine development. In this case, the vaccine is of interest for application in animals in first instance, but developing a vaccine against a pathogen of concern for animals that may in turn be a source of infection for humans is another strategy that may help in the fight against antimicrobial resistance. 

Lastly, Costanzo and Roviello (contribution 5) provide an overview of the state of the art regarding vaccines under development against the most concerning antibiotic-resistant bacteria and future directions to improve their development. First, the causes of antibiotic resistance and challenges in developing new antibiotics are outlined. Then, the authors elaborate on the rationale for implementing vaccination to fight antimicrobial resistance. Subsequently, they present a summary of current achievements on vaccines under development using these pathogens, including both those at the preclinical and clinical phases. Future strategies to improve vaccine efficacy, including bioconjugation, reverse vaccinology, and novel adjuvants, are also discussed. Altogether, this article offers a comprehensive review of the potential role of vaccines in preventing antimicrobial resistance.

Taken together, the articles in this Special Issue present preclinical studies of vaccines against bacterial pathogens of concern for public health and provide some clues regarding the future steps toward clinical studies. Although this collection has a relatively broad scope, this series of contributions will be of interest to the research community involved in the development of vaccines for protection against antibiotic-resistant bacterial infections and may provide valuable insights for achieving this aim.

